# The Ease of Language Understanding (ELU) model: theoretical, empirical, and clinical advances

**DOI:** 10.3389/fnsys.2013.00031

**Published:** 2013-07-13

**Authors:** Jerker Rönnberg, Thomas Lunner, Adriana Zekveld, Patrik Sörqvist, Henrik Danielsson, Björn Lyxell, Örjan Dahlström, Carine Signoret, Stefan Stenfelt, M. Kathleen Pichora-Fuller, Mary Rudner

**Affiliations:** ^1^Department of Behavioural Sciences and Learning, Linköping UniversityLinköping, Sweden; ^2^Linnaeus Centre HEAD, Swedish Institute for Disability Research, Linköping UniversityLinköping, Sweden; ^3^Department of Clinical and Experimental Medicine, Linköping UniversityLinköping, Sweden; ^4^Eriksholm Research Centre, Oticon A/SSnekkersten, Denmark; ^5^Department of Audiology/ENT and EMGO+ Institute for Health and Care Research, VU University Medical CenterAmsterdam, Netherlands; ^6^Department of Building, Energy and Environmental Engineering, University of GävleGävle, Sweden; ^7^Department of Psychology, University of TorontoToronto, ON, Canada; ^8^The Toronto Rehabilitation Institute, University Health NetworkToronto, ON, Canada; ^9^The Rotman Research Institute, Baycrest HospitalToronto, ON, Canada

**Keywords:** working memory capacity, speech in noise, attention, long-term memory, hearing loss, brain imaging analysis, oscillations, language understanding

## Abstract

Working memory is important for online language processing during conversation. We use it to maintain relevant information, to inhibit or ignore irrelevant information, and to attend to conversation selectively. Working memory helps us to keep track of and actively participate in conversation, including taking turns and following the gist. This paper examines the Ease of Language Understanding model (i.e., the ELU model, Rönnberg, 2003; Rönnberg et al., 2008) in light of new behavioral and neural findings concerning the role of working memory capacity (WMC) in uni-modal and bimodal language processing. The new ELU model is a meaning prediction system that depends on phonological and semantic interactions in rapid implicit and slower explicit processing mechanisms that both depend on WMC albeit in different ways. It is based on findings that address the relationship between WMC and (a) early attention processes in listening to speech, (b) signal processing in hearing aids and its effects on short-term memory, (c) inhibition of speech maskers and its effect on episodic long-term memory, (d) the effects of hearing impairment on episodic and semantic long-term memory, and finally, (e) listening effort. New predictions and clinical implications are outlined. Comparisons with other WMC and speech perception models are made.

## Background

### Overview

Some 30 years ago, we began a program of research to investigate the factors related to individual differences in speechreaders' ability to understand language. The findings underscored the importance of working memory capacity (WMC) for explaining those individual differences. In subsequent research, we extended our investigations to examine the associations between WMC and language understanding in other conditions, with the most recent focusing on audio-only speech understanding in adverse listening conditions by listeners using hearing aids.

The Ease of Language Understanding model (i.e., the ELU model, Rönnberg, 2003; Rönnberg et al., 2008) was developed, tested, and refined in an attempt to specify the role of working memory (WM) in a wide range of conditions in which people with normal or impaired hearing understand language. The language signal may be uni-modal or bi-modal speech or sign language and background conditions are realistic but provide contextual support or environmental challenge to differing degrees.

### Speechreading as compensation for hearing loss

Hearing loss leads to poorer perception of auditory speech signals and greater reliance on visual information available from the talker's face. Thus, we hypothesized, initially, that daily practice in visual speechreading by individuals with profound hearing loss or deafness would lead to superior, *compensatory* speechreading or speech understanding skills in comparison to normally hearing peers. One of the findings that motivated this hypothesis was that visual speechreading ability varies enormously between individuals (see Rönnberg, 1995 for a review). To test this hypothesis, we conducted several studies of speechreading in well-matched groups of individuals with normal hearing, moderate hearing loss and profound hearing loss. Contrary to the prediction, there were no significant group differences and thus no evidence of compensation for hearing loss by better use of visual speech information. Results were similar irrespective of type of presentation (video vs. real-life audiovisual; Rönnberg et al., 1983), type of materials (digits vs. discourse; Rönnberg et al., 1983), for just-follow conversation tasks (Hygge et al., 1992), for different durations of impairment, and for different degrees of hearing loss (e.g., Lyxell and Rönnberg, 1989; Rönnberg, 1990; Rönnberg et al., 1982, 1983). Spontaneous compensation for hearing loss through speechreading seemed, therefore, to be a cherished myth (Rönnberg, 1995).

### Perceptual and cognitive skills

These data prompted us to look for other ways to try to explain at least parts of the large variability in speech understanding observed across individuals (Rönnberg et al., 1998a, for an overview). In a set of studies, we identified the following predictor variables: verbal inference-making (sentence completion, Lyxell and Rönnberg, 1987, 1989), context-free word decoding (Lyxell and Rönnberg, 1991), and information processing speed that relies on semantic long-term memory (LTM; e.g., lexical access speed, Rönnberg, 1990; as well as rhyme decision speed; Lyxell et al., 1994; Rönnberg et al., 1998b). Indirect predictors of sentence-based speechreading performance included the VN 130/P200 peak-to-peak amplitude measure in the visual evoked potential (Rönnberg et al., 1989); WMC measured by the reading span test (Lyxell and Rönnberg, 1989; Pichora-Fuller, 1996); and verbal ability (Lyxell and Rönnberg, 1992). Overall, the indirect predictors were found to be related to sentence-based speechreading *via* their relationships with the direct predictors. This set of results demonstrated that WMC is strongly related to verbal inference-making, which in its turn is related to speechreading skill (Lyxell and Rönnberg, 1989); the amplitude of the visual evoked potential is related to speechreading via word decoding (Rönnberg et al., 1989); and verbal ability is related to speechreading via its relation to lexical access speed (Lyxell and Rönnberg, 1992).

### Other modalities of communication

A more general picture emerged as evidence accumulated that many of the predictor variables also related to other forms of communication. Successful visual-tactile speech communication and cued speech (i.e., a phonemic-based system which uses hand shapes to supplement speechreading) are predicted by phonological skills (e.g., Leybaert and Charlier, 1996; Bernstein et al., 1998; Leybaert, 1998). The precision of a phonological representation assessed by text-based rhyme tests has been shown to be an important predictor of the rate of visual-tactile (Rönnberg et al., 1998b; Andersson et al., 2001a,b), and visual speech tracking (Andersson et al., 2001a,b). In the same vein, audio-visual speech understanding in cochlear implant (CI) users is predicted by both phonological ability and individual differences in WMC measured using a reading span test (Lyxell et al., 1996, 1998).

### Importance of WM

Thus, about a decade ago, the data were pointing to an important role for WMC in predicting, directly or indirectly, the individual differences in speech understanding in one or more modalities. Testing participants who were hard-of-hearing or deaf provided clues as to how to re-conceptualize theories concerning speech understanding in individuals with normal hearing to take into account how their performance varies across a continuum from ideal to adverse perceptual conditions. However, more direct tests of the hypothesis concerning the importance of WMC for speech understanding in atypical cases and conditions were needed.

## Specifying the role of WMC in ease of language understanding

### Defining WM

WM is a limited capacity system for temporarily storing and processing the information required to carry out complex cognitive tasks such as comprehension, learning, and reasoning. An individual's WMC, or span, is measured in terms of their ability to simultaneously store and process information. Importantly, *complex* WMC is the crucial ability when it comes to understanding language, viz being able to store *and* process information relatively simultaneously. Simple span tests, such as digit span, mainly tap storage functions in short-term memory, and tend not to be such good predictors of language comprehension, reading ability and speechreading ability [for an early review see Daneman and Merikle (1996); but see Unsworth and Engle (2007)]. We have usually assessed WMC using a *reading* span test (Daneman and Carpenter, 1980; Rönnberg et al., 1989; Just and Carpenter, 1992). In the reading span test procedure, the participant reads a sentence as quickly as possible and then performs a task to ensure that the sentence has been fully processed. After a small set of sentences has been presented, read and understood, the participant is asked to recall either the first or last word of each of the sentences in the set in the order in which they were presented. Set size gradually increases and the WM span is determined to be the largest set size for which the individual can correctly recall a minimum specified proportion of the words. We have found in our research that the total number of words correctly recalled in any order, is a more sensitive predictor variable than set size (Rönnberg et al., 1989; Lunner, 2003; Rönnberg, 2003). The basic assumption is that, as the processing demands of the reading span task increase, there will be a corresponding decrease in how much can be stored in the limited capacity WM system. Total reading span score is used to gauge this trade-off between WM processing and storage.

There is a strong cross-modal relationship between reading span scores (visual-verbal) and spoken communication skills (auditory-verbal), implying that it is supported by a *modality-general* ability (cf. Daneman and Carpenter, 1980; Just and Carpenter, 1992; Kane et al., 2004). This may explain why WMC has a predictive power that applies to several communicative forms (e.g., Ibertsson et al., 2009). Moreover, the reading span test (and other similar complex span tests) seems to tap into semantic *processes* such as inhibition of irrelevant information (in particular inhibition of context-irrelevant word-meaning; Gunter et al., 2003), the ability to *selectively attend to* one channel of information (Conway et al., 2001), the ability to *divide attention* between channels (Colflesh and Conway, 2007), and the ability to *store* and integrate signal-relevant prior *semantic* cues (Zekveld et al., 2011a, 2012). The similiarity of results across the wide range of conditions applied in these studies supports the role of WMC in on-line language processing. Our research assessed the role of WMC during language understanding in various conditions such as when speech is processed visually by speechreaders, when auditory speech is heard in noise, by listeners with hearing impairment, when hearing aids are used, and when sign rather than speech is the signal used to convey language.

### Extreme speechreading skill

Case studies of extremely skilled speechreaders (Rönnberg, 1993; Lyxell, 1994; Rönnberg et al., 1999) demonstrated that bottom-up processing skills (e.g., lexical access speed and phonology) are only important up to a certain *threshold*, or level of language understanding. The threshold is assumed to be due to the efficiency of phonologically mediated lexical access, constrained by neural speed at different levels in the perceptual-cognitive system (Pichora-Fuller, 2003). We showed that to surpass this threshold, and to become speechreading experts, the individual has to be equipped with large complex WMC and related verbal inference-making and/or executive skills (see also Andersson and Lidestam, 2005). This seemed to be true irrespective of communicative habit—participant GS used tactile speechreading (Rönnberg, 1993), participant MM was bilingual in sign and speech (Rönnberg et al., 1999), and participant SJ used visual speechreading strategies only (Lyxell, 1994). The effects could not be explained in terms of age, degree of hearing loss, or even onset of the loss.

### Noise, hearing loss, and hearing aids

The importance of predictions of individual differences in speech understanding based on reading span was crucially demonstrated when a strong association was found with spoken sentence recognition in noise by individuals with hearing loss irrespective of whether they were tested with or without hearing aids (Lunner, 2003). During conversation, the individual who has impaired hearing must orchestrate the interplay between distorted perceptual input, LTM, and contextual cues. We argue that the storage and processing abilities reflected in complex WMC tasks are essential for such compensatory interactions in people with hearing loss. WMC also seems to play an important role when people with normal hearing must understand language spoken in acoustically adverse conditions (for discussions see Mattys et al., 2012; Pichora-Fuller et al., 1995; McKellin et al., 2007).

### Sign language

Insight into the role of WM in sign language communication also led to a series of studies at our lab investigating the neurocognitive mechanisms of WM for sign language (Rönnberg et al., 2004; Rudner et al., 2007, 2010, 2013; Rudner and Rönnberg, 2008a,b). These studies demonstrate similar neurocognitive mechanisms across language modalities with some modality-specific aspects. These language modality-specific differences include a greater involvement of superior parietal regions in WM for sign language and a de-emphasis of temporal processing mechanisms.

Taken together, the speech understanding and sign language findings set the stage for formulating the Ease of Language Understanding model (ELU, see Rönnberg, 2003; Rönnberg et al., 2008) to extend existing more general models of WM in order to account for a wide range of communication conditions.

## The original WM system for *E*ase of *L*anguage *U*nderstanding (ELU)

The broader context of the ELU model is that of cognitive hearing science. Cognitive Hearing Science is the new field that has emerged in response to general acknowledgement of the critical role of cognition in communication (Arlinger et al., 2009). Characteristic of cognitive hearing science models is that they emphasize the subtle balancing act, or interplay between bottom-up and top-down aspects of language processing (e.g., Schneider et al., 2002; Scott and Johnsrude, 2003; Tun et al., 2009; Mattys et al., 2012). The ELU model describes how and when WM is engaged to support listening in adverse conditions, and how it interacts with LTM. In the original version we did not distinguish between episodic and semantic LTM but subsequent research and theoretical development have proven that this is an important distinction (see under EXTENDING THE ELU APPROACH:.). Episodic memory is memory of personally experienced events (tagged by time, place, space, emotion and context, see Tulving, 1983). Semantic memory refers to general knowledge, without personal reference (e.g., vocabulary and phonology).

**Figure 1:**
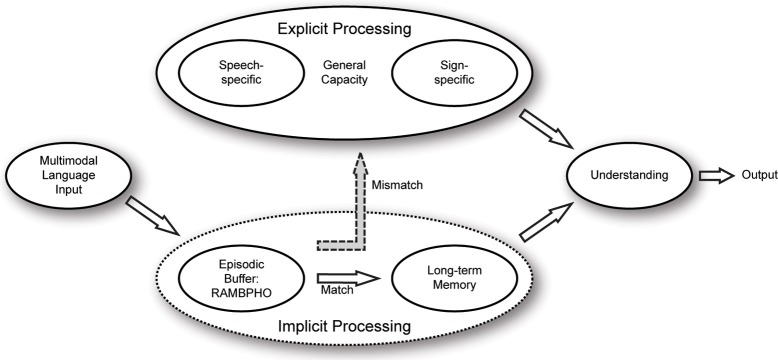
**The working memory model for Ease of Language Understanding (ELU, adapted from Rönnberg et al., 2008)**.

In the original model (see Figure 1; Rönnberg, 2003; Rönnberg et al., 2008; Stenfelt and Rönnberg, 2009), we assumed that multimodal speech information is Rapidly, Automatically, and Multimodally Bound into a PHOnological representation in an episodic buffer (cf. Baddeley, 2000, 2012) called RAMBPHO. RAMBPHO is assumed to operate with syllables that feed forward in rapid succession (cf. Poeppel et al., 2008; Bendixen et al., 2009). If the RAMBPHO-delivered sub-lexical information matches a corresponding syllabic phonological representation in semantic LTM, then lexical access will be successful and there is no need for top-down processing. And, if RAMBPHO continues to provide matching syllabic information, lexical retrieval will continue to occur implicitly and at a rapid rate. The time-window for the assembly of the RAMBPHO information and for successful lexical retrieval is assumed to start when activation begins at a cortical level [superior temporal gyrus (STG)/posterior superior temporal sulcus; (Poeppel et al., 2008)], where the neural binding of syllabic auditory and visual speech seems to occur (around 150 ms after speech onset, Campbell, 2008), and then it generally takes another 100–250 ms before lexical access presumably occurs supported by neural mechanisms in the left middle temporal gyrus(MTG)/inferior temporal gyrus (Poeppel et al., 2008; see also Stenfelt and Rönnberg, 2009). If, however, the RAMBPHO information cannot be immediately related to phonological representations in semantic LTM or is not precise enough to match them unambiguously, lexical access is delayed, temporarily disrupting the feed-forward cycle of information flow. Explicit and deliberate WM processes are assumed to be invoked to compensate for this mismatch between RAMBPHO output and LTM representation. These explicit processes typically operate on another time scale, measured in seconds rather than milliseconds (Rönnberg et al., 2008). Examples of such processes include inference-making, semantic integration, switching of attention, storing of information, and inhibiting irrelevant information. While the source of the mismatch is at the lexical level, later explicit compensation may involve other linguistic levels. WMC is assumed to be required for most explicit processing aspects/subskills.

Generally then, depending on the conditions under which the incoming speech signal unfolds (ambient noise, hearing impairment, signal processing in the hearing aid, etc.), as well as the precision and quality of the semantic LTM representation, the relative contributions of explicit and implicit processes will continuously fluctuate during a dialogue.

## Initial tests of the model

The experimental studies performed to test the model have principally used two types of manipulation, one based on hearing aid signal processing and one based on presentation of text cues in order to induce a mismatch between RAMBPHO and semantic LTM. Initial testing was done primarily with spoken language presented in auditory noise, but the model is also likely to be applicable to signed languages presented in visual noise (cf. Speranza et al., 2000). Brain imaging studies (e.g., Söderfeldt et al., 1994; Rönnberg et al., 2004; Rudner et al., 2007, 2013; Cardin et al., 2013) suggest that similar but not identical neural networks are active for processing sign language and speech, and that the close relation between semantics and phonology in sign language may influence the mismatch mechanism (Rudner et al., 2013).

### Using auditory signal processing manipulations to induce phonological mismatch

Wide Dynamic Range Compression (WDRC) is one of the technologies used in modern digital hearing aids to increase speech intelligibility by applying non-linear amplification of the incoming signal such that soft sounds become audible without loud sounds becoming uncomfortable. However, this non-linear signal processing can also have side-effects that distort the phonological properties of speech, especially when compression release is fast. We used this phenomenon to investigate the main prediction of WM-dependence in the ELU model in experienced hearing-aid users. Hearing aids were experimentally manipulated such that participants received WDRC for the first time. According to the model, given that a syllabic segment in the speech stream is processed with a new algorithm, the sound may seem different compared to the one delivered by the habitual algorithm, thus causing a relative RAMBPHO-induced mismatch with the phonological-lexical representation in LTM. Results showed that individual differences in WMC accounted for most of the variance in the threshold for 50% correct word recognition on speech-in-noise tests, irrespective of whether stationary or modulated noise backgrounds were applied (Foo et al., 2007; cf. Desjardins and Doherty, 2013). This means that as long as we disrupt the habitual processing mode, WMC is invoked.

A follow-up intervention study was conducted to investigate how the relationship between WMC and mismatch might change as the individual acclimatized to a new hearing aid algorithm. Again, participants who were habitual hearing aid users were switched to a new fast or slow signal processing algorithm in the hearing aid. After nine weeks of experience with one kind of hearing aid compression, participants were tested either with the same kind of compression (“matching” conditions), or with the other kind of compression of which they had no experience (“mismatching” conditions). As predicted, in one study conducted in Swedish (Rudner et al., 2009a,b) and in another conducted in Danish (Rudner et al., 2008), thresholds for 50% correct word recognition on speech-in-noise tests for mismatching compression conditions were correlated with WMC. WMC was not the main predictor of speech-in-noise thresholds for matching conditions. Independent studies support the notion that WMC is crucial to speech understanding in adverse conditions by hearing aid users (Gatehouse et al., 2003, 2006; Lunner, 2003; Akeroyd, 2008; Rudner et al., 2011; Mattys et al., 2012).

Using the visual letter monitoring task as an index of WMC, Lunner and Sundewall Thorén (2007) showed that WMC accounted for about 40% of the variance in the ability to perceive speech in modulated background noise with FAST compression. Pure-tone average hearing loss, on the other hand, accounted for less than 5% of the variance. Importantly, the pattern was reversed when compression by the hearing aid was SLOW and tests were conducted in steady-state noise conditions: WMC explained only 5% of the variance while pure-tone hearing loss explained 30%. Lunner and Sundewall Thorén (2007) suggested that FAST compression in modulated noise backgrounds better reflects the more rapid changes in the signal and noise characteristics of everyday listening conditions. Hence, using SLOW compression in steady-state noise conditions may underestimate everyday cognitive demands (cf. Festen and Plomp, 1990). The conclusions drawn in the studies using WDRC are consistent with recent findings in which other advanced hearing aid signal processing algorithms were used: Arehart et al. (2013) found that a high degree of frequency compression reduced intelligibility more for individuals with low WMC compared to individuals with high WMC, especially for older adults.

The emerging picture seems to be that advanced signal processing algorithms designed to improve intelligibility and listening comfort may also generate RAMBPHO-dependent mismatch due to distortions at the syllable level caused by unfamiliar amplitude or frequency compression. Thus, there is a benefit and a cost from such signal processing. Mismatches, or costs, are overcome more successfully by individuals with high WMC.

### Using textual manipulations to create phonological mismatch

Severe hearing loss can lead to phonological deterioration in semantic LTM (Andersson, 2002; Lazard et al., 2010; Rönnberg et al., 2011b). Classon et al. (2013a) undertook a study that tested the hypothesis that high WMC can compensate for poor phonological skills in individuals with hearing impairment. To avoid audibility problems, phonological mismatch was manipulated using text rather than speech. Classon et al. (2013a) showed that hearing impairment negatively affected performance on a text-based task in which participants decide if two words rhyme or not in RAMBPHO-dependent, mismatching conditions. Mismatch was created in conditions where the two test words rhymed but were orthographically dissimilar, or alternatively, did not rhyme but were orthographically similar (Lyxell et al., 1993, 1998; Andersson and Lyxell, 1998; Andersson, 2002). In the latter case, orthographic similarity may induce an incorrect “yes” response when words do not rhyme, if the phonological precision of representations in semantic LTM is compromised. The prediction based on the ELU model is that participants who have a high WMC will be able to compensate for poor phonological representations because they can keep representations in mind and double-check back and forth to ensure that the words really do not rhyme before they decide. The data confirmed this prediction. Hearing impaired participants with high WMC performed on a par with normal hearing participants, whereas hearing impaired participants with low WMC displayed higher error rates than the normal hearing subgroups with low WMC. Note that hearing impairment did not confound the results since the level of WMC was matched across groups with normal hearing and hearing impaired participants, and there was no difference in the degree of hearing impairment between the high vs. low WMC subgroups.

### Semantic strategy in rhyme tasks

The effects of hearing impairment on the mismatching conditions in the yes/no rhyme task may be attributed to imprecise phonological representations in semantic LTM. This may lead automatically to a non-phonological orthographic bias, and perhaps even a semantic bias, when written words are presented in a rhyme task, especially for individuals with hearing impairment who have a low WMC. The plausibility of such an explanation was reinforced by the finding that participants with low WMC outperformed participants with high WMC on subsequent incidental recognition of items that had been correctly identified in the initial rhyme testing phase. Since semantic processing has been shown to promote episodic LTM (e.g., Craik and Tulving, 1975), a semantic interpretation of this seemingly paradoxical result may fall into place.

Connected to this semantic interpretation of the rhyming data, is the fact that the test of WMC that we have been discussing so far, the reading span test, also measures important semantic interpretation processes. Although the semantic absurdity judgments typically demanded in this task (Rönnberg et al., 1989) were initially introduced to ascertain that the participants actually processed the whole sentence rather than strategically focusing only on the first or final words, semantic processing may in itself be an important component of the test. Indeed, sentence completion ability (tapping semantic integration and grammar) under time pressure is significantly correlated to performance in the reading span test (Lyxell and Rönnberg, 1989). Although the reading span test taps into several storage and processing components summarized by one measured variable, a semantic perspective on reading span may cast new light on old data in that rapid sense-making and semantic judgment is demanded in the reading span test as well as in the sentence-completion task.

### Neural signatures of text-speech semantic mismatch

WMC, again measured with the reading span task, has in recent studies been shown to modulate the ability to use semantically related cues and to suppress unrelated, “mismatching” cues to help understand speech in noise (Zekveld et al., 2011a, 2012). Interestingly, both the WMC of the participants and the lexicality of text cues modulated neural activation in the left inferior frontal gyrus (LIFG) and the STG during speech perception. Presumably, these areas are related to compensatory processes in semantic cue utilization. Independent data also suggest that LIFG is involved in semantic and syntactic processing networks (Rodd et al., 2010). Cortical areas beyond the temporal lobe are engaged in the processing of intelligible but degraded speech (Davis and Johnsrude, 2007). It is quite plausible that there is a functional connectivity between LIFG and STG, and that LIFG modulates STG via top-down connections when semantic processing is involved (Obleser and Kotz, 2011). In fact, the general picture is that there are ventral and dorsal pathways that connect pre-frontal and temporal language-relevant regions which support semantic and syntactic processes (Friederici and Gierhan, 2013).

## Interim summary

Thus far, we can infer the architecture of a WM system (i.e., the ELU model) that is invoked when there is some kind of signal processing that changes the phonological structure of the speech signal, or when there is a combination of signal processing and fluctuating background noise that puts large demands on phonological processing. In addition, there is also new evidence to suggest that a semantic mismatch requires WM resources to help focus on the target-speech signal while inhibiting distracting semantic cue information as will be discussed further below.

## Extending the ELU approach: WMC related to attention, memory systems, and effort

In the following sections, evidence is reviewed indicating that WMC plays a part in (a) “early” attention processes, (b) short-term retention of spoken information when the signal is processed by hearing aids, (c) inhibition and episodic LTM of masked speech, (d) the effects of hearing impairment on episodic and semantic LTM, and (e) listening effort. These data—behavioral and physiological—have shaped a new version of the ELU model, which will be presented subsequently.

### WMC influences early attentional processes

This section suggests that high WMC is associated with neural interactions that facilitate attention and which are important for further speech signal processing (Peelle and Davis, 2012). This kind of cognitive tuning of the brain does not seem to involve any explicit processing component, although it is dependent on WMC.

WMC is related to the ability to inhibit processing of irrelevant information and overrule undesired but pre-potent responses (e.g., Kane et al., 2001; Engle, 2002). More precisely, high-WMC individuals appear to have a superior ability to modulate attention span (i.e., how much information that has access to cognitive processing). Where in the processing chain filtering out of irrelevant information takes place is still a subject of debate. Relations between WMC and early cortical auditory processing (as reflected in the amplitude of the N1 component of event-related potential measures) have been demonstrated with greater amplitudes for attended sound and lesser amplitudes for ignored sound in high-WMC individuals (Tsuchida et al., 2012). However, a recent experiment in our lab (Sörqvist et al., 2012a) suggests that WMC is involved in filtering processes at even earlier (sub-cortical) stages. Normally-hearing participants visual-verbal performed a visual *n*-back (1-, 2-, 3-back) task (Braver et al., 1997) while being presented with to-be-ignored background sound. In a control condition, the participants just heard the sound and did not perform any task. In the *n*-back task, WM load increased with increasing *n* and the control condition represented least load. The magnitude of the auditory brain stem response (ABR, wave V, on average 7 ms post-stimulus onset) was negatively associated with WM load. Moreover, higher WMC scores were related to a greater difference of the ABR between conditions. Thus, both the experimental load manipulation and correlational evidence converge on the same conclusion: early attentional processes interact with WM. Our interpretation is that cognitive load reduces resources at the peripheral level, and the relation with WMC suggests a relationship between central and peripheral capacity.

One mechanism underpinning this relation might be the alpha rhythm. Alpha rhythms reflects the cognitive system's pre-stimulus preparation for incoming stimuli, enabling efficient processing (Babiloni et al., 2006), and have been associated with both WM load *and* processing of acoustically degraded stimuli (Obleser et al., 2011). Moreover, in a recent focused review of brain oscillations and WM, it was suggested that the alpha rhythm serves as an attentional gate-keeper to optimize the signal-to-noise ratio for WM-based processing, and that the number of gamma cycles that fit within one theta cycle may index WMC (Freunberger et al., 2011).

However, single indices may only tell part of the story of how brain oscillations relate to WM. In a recent review, it has been argued that the correlations between oscillatory phases in different brain regions, so called phase synchronization, affect the relative timing of action potentials. This is important for a memory system such as WM, which in turn depends on the interaction between different storage and executive processing components (and their corresponding phases), for example, phase correlations between pre-frontal and temporal regions (Fell and Axmacher, 2011).

Thus, a high WMC may facilitate neural fine-tuning at an early level of auditory processing (cf. Pichora-Fuller, 2003; Sörqvist et al., 2012a) but may also reflect a highly synchronized brain network (Fell and Axmacher, 2011). The conclusion about some kind of fine-tuning is further reinforced by the finding that WM processes are interconnected with the effects of practice on auditory skills (Kraus and Chandrasakaren, 2010) and their corresponding neural signatures (Kraus et al., 2012).

All in all then, data from independent labs suggest that WMC is related to several brain oscillation indices, and that WMC is related to early attention processes. This WMC-based top-down influence on speech-relevant attention processes may be part of the explanation as to why attending to a speaker in a multi-talker situation gives rise to dedicated neural representations (Mesgarani and Chang, 2012).

### WMC interacts with signal processing and short-term retention

This section presents data showing for the first time that hearing aid signal processing can improve short-term memory in hearing-impaired individuals, and that this effect is modulated by WMC (Ng et al., 2013a,b). This may prove to have important clinical consequences (Piquado et al., 2012).

Even when audibility is controlled (e.g., by amplifying speech with hearing aids), individuals with hearing impairment still perform worse than young normal-hearing subjects, with cognitive factors accounting for residual variance in performance (e.g., Humes, 2007). Attentional resources may contribute to speech understanding, especially in effortful or divided attention tasks (Tun et al., 2009; Rönnberg et al., 2011a,b). For example, Tun et al. (2009) have shown poorer delayed recall for audible auditory stimuli in participants with impaired compared to normal hearing when encoding took place under divided attention conditions. Rönnberg et al. (2011a,b) also demonstrated that short-term memory performance under divided attention encoding conditions correlated with degree of hearing impairment (cf. Humes et al., 2006).

Hearing aid signal processing schemes may reduce attention costs while listening to speech in noise and thus improve speech understanding. It has been demonstrated that noise reduction signal processing reduces listening effort for people with normal hearing (Sarampalis et al., 2009). In a recent study (Ng et al., 2013a,b), we examined how hearing aid signal processing influences word recall in people with *hearing impairment*. The scheme under investigation was binary time-frequency masking noise reduction (Wang et al., 2009). Each participant listened to sets of eight sentences from the Swedish Hearing-In-Noise-Test (HINT) materials (Hällgren et al., 2006) in 4-talker babble or stationary noise, with and without noise reduction. To control audibility, SNRs were individualized such that performance levels were around 95% for word recognition in stationary noise with individual linear amplification and individually prescribed frequency response. Typical SNRs for 95% correct were around +5 dB. Each participant recalled as many sentence-final words as possible after each set of sentences had been presented. We found that participants performed worse in noise than in quiet and that this effect was partially restored by noise reduction. In particular, individuals with high WMC recalled significantly more of the items from the end of the lists (recency position) presented in noise when noise reduction was used.

Thus, WMC interacts with signal processing in hearing aids and facilitates short-term memory. There is obviously room for improvement even when the audibility of the signal is good, a fact that offers a new perspective on how to conceptualize benefits from different kinds of signal processing in hearing aids.

### WMC—especially the inhibitory aspects—determine episodic LTM for prose masked by speech

This section is about how WMC relates to inhibition of an interfering talker during listening to sentences and to later long-term episodic recall.

We have recently shown that WMC seems to be related to long-term retention of information that is conveyed by masked speech (Sörqvist et al., 2012b). Young, normally-hearing students listened to invented stories (each about 7.5 min long) about fake populations and afterwards answered questions about their content (e.g., what did the lobiks wear in the kingdom of death?). The stories were spoken in a male voice and masked by another male voice (normal or spectrally-rotated; Scott et al., 2009).

Two types of complex WMC tests were administered separately: the reading span and the size-comparison (SIC) span test (Sörqvist et al., 2010). The SIC span is a WMC test that targets the ability to resist semantic confusion. It involves comparing the size of objects while simultaneously maintaining and recalling words taken from the same semantic category as the to-be-compared words. The distinguishing feature of the test is that the semantic interference between the comparison words and the to-be-recalled words must be resolved by inhibiting the potential semantic intrusions from the comparison words.

Ability to answer content questions was superior when the story was masked by a rotated as compared with a non-rotated speech signal. More importantly, SIC span was a better predictor variable than reading span of the magnitude of this difference (Sörqvist et al., 2012b). We argue that the inhibition ability tapped by SIC span is involved during resolution of the confusion between competing and target speech and that better resolution enhances episodic encoding and retrieval. This will, at least in part, determine an individual's ability to remember the important parts of a conversation.

Speech-in-speech processing studies have typically addressed speech perception as such (e.g., Bronkhorst, 2000). Our contribution is that we associate WMC—and the inhibition component in particular—with the encoding carried out during speech-in-speech comprehension, and how this type of WMC encoding relates to episodic LTM (cf. Hannon and Daneman, 2001; Schneider et al., 2010). There is some evidence of a relation between episodic LTM and cognitive spare capacity (Rudner et al., 2011; Mishra et al., 2013).

### Degree of hearing impairment in hearing aid users is associated with episodic LTM

This section summarizes a recent cross-sectional study on a sample of hearing aid users and how their hearing thresholds are associated with the efficiency of different memory systems.

Despite the possibility of using hearing aids, hearing problems continue to occur in everyday listening conditions. Many people who own hearing aids do not use them on a regular basis. For those who do wear them regularly, signal processing algorithms in hearing aids cannot generally provide an optimal listening situation in noisy and challenging conditions (Lunner et al., 2009). By including hearing aid users (*n* = 160) from the longitudinal Betula study of cognitive aging (Nilsson et al., 1997), we made a conservative test of the hypothesis that hearing impairment is negatively related to episodic LTM deficits. The basis of the prediction from the ELU model (Rönnberg et al., 2011a,b) is that mismatches will remain despite the use of a hearing aid, and hence fewer items will be encoded and retrieved from episodic LTM. Therefore, we assume a *disuse* effect on episodic LTM, leading to a less efficient episodic memory system. However, short-term memory (STM, here operationalized by Tulving and Colotla, 1970; the Tulving and Colotla lag measure) and WM (not explicitly measured in this study) should be increasingly active in mismatching conditions because both systems would be constantly occupied during retrospective disambiguation of what had been said in a conversation. Thus, both STM and WM would be *relatively less vulnerable to disuse*. It is also predicted that semantic LTM should be highly correlated with episodic LTM because the status of phonological representations in semantic LTM should be tightly related to the success of encoding into episodic LTM. These predictions have recently been confirmed by structural equation modeling. Episodic LTM decline is related to long-term hearing impairment, despite the use of existing hearing aid technology (Rönnberg et al., 2011a,b). One note of caution though is that exact measures of every-day hearing aid use were not available. Hence, any potential dose-response relationship among the hearing aid wearers could not be assessed.

Thus, hearing loss was independently related to episodic LTM (verbal recall tasks) and semantic LTM (initial letter fluency and vocabulary) but unrelated to STM, even when age was accounted for. Visual acuity alone, or in combination with auditory acuity, did not contribute to any acceptable structural equation model; it only made the prediction of episodic LTM decline worse by standard goodness-of-fit criteria (see also Lindenberger and Ghisletta, 2009). And finally, even when the episodic LTM tasks were of non-auditory nature (i.e., motor encoding of lists of imperatives and subsequent free recall of these actions, Nilsson et al., 1997) the association with hearing loss persisted (Rönnberg et al., 2011a,b).

Although the participants wore their hearing aids whilst completing the auditory episodic memory tasks, this negative result may be accounted for in terms of perceptual stress, or information degradation (cf. Pichora-Fuller et al., 1995). It has been argued and empirically demonstrated that once perceptual stress is equated for example among different age groups, differences in performance on WM, associative memory and comprehension tasks (e.g., Schneider et al., 2002) tend to vanish. Nevertheless, the decreased performance in the non-auditory tasks reported in Rönnberg et al. (2011a,b) cannot be explained on the basis of information degradation and it is possible that there are both information degradation and long-term deprivation effects. Central mechanisms involving attentional resources could also be affected by hearing impairment, which in turn would predict problems with memory encoding (Tun et al., 2009; Majerus et al., 2012; Peelle and Davis, 2012; cf. Sörqvist et al., 2012a) and possibly WMC (see also Schneider et al., 2010). Before we can reach definite conclusions about the selective effects of hearing impairment on memory systems, a broader spectrum of tasks assessing different memory systems must be employed.

From a more general and clinical perspective, we suggest that future longitudinal studies should evaluate the effects of the use of the hearing aids on cognition and memory systems, and in particular, the effects of certain kinds of signal processing on different tasks assumed to index different memory systems.

### WMC and effort

In this section, we discuss recent work related to the ELU prediction about WMC and effort (cf. Hervais-Adelman et al., 2012; Amichetti et al., 2013). In particular, we focus on predictions based on recent data using pupillometry that contrast with the ELU prediction.

Apart from taxing cognitive capacity, listening under adverse conditions is often associated with subjectively experienced effort, especially in individuals with hearing impairment (Pichora-Fuller, 2006). The ELU prediction about effort, or the inverse notion of “ease” (Rönnberg, 2003) is that in effort-demanding listening situations, an individual with a high WMC will be better able to compensate for the distorted signal, without exhausting WMC and therefore experience less effort in comparison to an individual with small WMC (cf. the neural efficiency hypothesis; e.g., Pichora-Fuller, 2003; Heitz et al., 2008), given that the task does not hit ceiling/floor (Rönnberg, 2003). Intermediate difficulty levels provide the best opportunity for explicit processes to operate in a compensatory fashion. Recent work by our group has confirmed that higher WMC is associated with lower perceived and rated listening effort for intermediate levels of difficulty, or “ease” of processing (Rudner et al., 2012). We suggest that subjective effort ratings may be useful for understanding the relative contributions of explicit WM processes to speech understanding in challenging conditions (Rudner et al., 2012; Ng et al., 2013b).

Some researchers have proposed that the pupillary response reflects cognitive processing load during the processing of sentences of different grammatical complexity (Piquado et al., 2010; Zekveld et al., 2010). This response is also sensitive to age, hearing loss, and the extra effort required to perceive speech in competing talker conditions compared to noise maskers (Zekveld et al., 2011b; Koelewijn et al., 2012a). Koelewijn et al. (2012b) observed that people with high SIC spans demonstrated larger pupil size, and that higher SIC span performance, in turn, was related to lower signal-to-noise ratios needed to perform at a certain threshold level in the competing talker condition. This pattern of findings may suggest that cognitive load is actually increased by high WMC, which can be viewed as a paradoxical result, but has support in the literature (Van der Meer et al., 2010; Zekveld et al., 2011b). Another interpretation of these data is that individuals with a high capacity solve difficult stimulus conditions by consuming more cognitive brain resources (more extensively or more intensively), thus exercising greater task engagement, and this is what is reflected in the pupil size variations (Koelewijn et al., 2012b; see Grady, 2012).

Pupil size seems to reliably capture cognitive load and associated effort under certain semantic or informational masking conditions. The exact mechanisms behind the empirical findings so far remain to be elucidated. But clinically, irrespective of explanatory mechanism, pupil size may become a complementary measure to subjective ratings of effort.

## General discussion and a new ELU-model

Phonological and semantic mismatches increase the dependence on WMC in speech-in-noise tasks. However, as we have seen in the current review of recent ELU-related WMC studies, the role of WMC is extended to include early attention mechanisms, interactions with memory systems under different multi-talker conditions, both for short-term and LTM, and a relationship to effort via subjective and objective measures.

Below we present the new empirical extensions that emerge from our recent data inspired by the old ELU model (Rönnberg et al., 2008). Then, we describe the new ELU model, based on these new empirical patterns, emphasizing in general and in detail the new features that differ from the old model. A section on predictions will close the presentation of the new model. In the following section, the new ELU model is compared to other relevant WM and speech perception models. The paper ends by addressing some important clinical consequences that follow from the model.

### New empirical extensions

First, the data we have presented and discussed suggest that several kinds of signal processing in hearing aids (*i.e., fast amplitude compression, frequency compression, and binary masking*), designed to facilitate speech perception, are handled best by individuals with high WMC. This is the first extension from the original studies that informed the development of the ELU model (Rönnberg et al., 2008). At that time, we did not know whether WMC was important for just one kind of distortion induced by signal processing (i.e., fast amplitude compression) or not. Importantly, when some kind of distortion of the signal is introduced, the feed-forward mechanism (cf. Bendixen et al., 2009) of RAMBPHO that predicts yet-to-be-experienced (syllabic) elements in the unfolding sound sequence (cf. Poeppel et al., 2008; Bendixen et al., 2009) seems to be temporarily interrupted, allowing ambiguous information to enter an explicit processing loop before understanding can be achieved.

A second extension is related to the *pre-tuning or synchronization* by WMC, directly or indirectly, prior to or early on during stimulus presentation. One type of prior influence mediated by WMC relates to “early” attention processes (Fell and Axmacher, 2011; Freunberger et al., 2011; Sörqvist et al., 2012a), another is related to priming, or pop-out (e.g., Davis et al., 2005). Recent data suggest that the magnitude of the pop-out effect may be mediated by WMC (Signoret et al., 2012). A third kind of influence exerted by WMC relates to memory encoding operations (Sörqvist and Rönnberg, 2012), and subsequent influences on understanding, including turn-taking in a dialogue (Ibertsson et al., 2009). This kind of continuous feedback was not part of the old ELU model (Rönnberg et al., 2008). This means that the new model also acknowledges a *post-dictive*, explicit feedback loop, feeding into *predictive* RAMBPHO processing. This mechanism is akin to the hypothesis testing, analysis-by-synthesis aspect of the Poeppel et al. (2008) framework (see below under Relation to other models).

A third extension has to do with the role of WMC in processing text cues that generate *explicit semantic expectations* of what will come in the unfolding speech stream. WMC is particularly important when expectations are violated by the content of the speech signal (Zekveld et al., 2011a). This may be because individuals with high WMC have a superior ability to inhibit the cue-activated, *mismatched* representation in semantic memory (cf. Nöstl et al., 2012; Sörqvist et al., 2012b). The discovery of a semantic influence on RAMBPHO processing means that the theoretical assumption of the model must be revised (see further below). Further, research suggests that older people more frequently rely on semantic context. For older people, incongruent semantic context seems to impair identification of words in noise, although confidence levels are higher than in younger adults (Rogers et al., 2012). Older people have a smaller WMC than younger individuals while frontal-lobe based executive functions may remain intact and this may account for the false hearing effects (Rogers et al., 2012). Also, over many decades of greater reliance on context in the face of gradual age-related declines in sensory processing, there may be changes in brain organization with an anterior-posterior shift in the brain areas engaged in complex tasks (Davis et al., 2008).

A fourth extension is that *high WMC individuals can deploy more resources to both semantic and phonological aspects* of a task, depending on instruction. The versatility in types of processing (phonological and semantic) of high WMC people represents a feature that was lacking in the old model. For example, in the Sörqvist and Rönnberg (2012) study WMC contributed to inhibition of a competing talker while focusing on the semantic content of the target talker. A consequence of this is enhanced, or deeper, understanding (Craik and Tulving, 1975). The by-product is more durable episodic memory traces (Classon et al., 2013a). In a recent ERP study Classon et al. (2013b) showed that hearing impaired, but not normal hearing individuals, demonstrate an amplified N2-like response in non-rhyming, orthographically mismatching conditions. This ERP signature of hearing impairment is suggested to involve increased reliance on explicit compensatory mechanisms such as articulatory recoding and grapheme-to-phoneme conversion and may prove to tap into some phonological WM function.

A fifth important extension encompasses the *negative relationships between hearing loss and episodic and semantic LTM*. These occur despite the use of hearing aids. However, STM is relatively unaffected, presumably because the demand to resolve speech understanding under mismatching, adverse conditions keeps this memory system in a more active state. Therefore, the overall selective effects on different memory systems are couched in terms of use/disuse (Rönnberg et al., 2011a,b). It should be noted that although the ELU prediction is in terms of relative effects of use/disuse, it does not exclude the possibility that either STM or WM may be affected by hearing impairment (cf. Van Boxtel et al., 2000; Cervera et al., 2009); it only predicts a relatively larger LTM impairment.

A sixth general fact to note is the *modality-generality* of memory systems in relation to language understanding. Reading span obviously taps modality-general verbal WMC as it predicts variance in the speech-in-noise tasks (Akeroyd, 2008; cf. Daneman and Carpenter, 1980; Just and Carpenter, 1992). Generality is also a key feature of the modulation of auditory attention (ABR) by manipulating visual-verbal WM load (Sörqvist et al., 2012a). Finally, a striking finding in the (Rönnberg et al., 2011a,b) study is that the negative memory consequences that may be attributed to hearing loss also show an independence of encoding format, and is not uniquely related to auditory encoding: At the level of simple correlations, hearing loss showed the highest negative correlation to free recall performance on tasks which not only involved auditory encoding but also encoding of motor and textual representations—and the effects were still manifest after statistically correcting for age.

Seventhly, and finally, the effect of WMC on stimulus processing is pervasive in terms of the *time window*: from early brain stem responses to encoding into episodic LTM. Thus, the above generalizations have set the stage for a more analytical and general formulation of the ELU model.

### Theoretical consequences

The new extensions result in a better specified ELU model that presents WM as the arena for interpretating the meaning of an ongoing dialogue. An individual with high WMC is more capable of using different levels/kinds of information and implicit/explicit strategies for extracting meaning from a message. The storage and processing operations that are performed by a high-capacity system are modality-general and flexible during multi-tasking. Implicit and explicit processes are assumed to run in parallel and interactively, but under different time windows (cf. Poeppel et al., 2008).

The successful listener disambiguates the signals on-line over time, due to successive semantic and lexical retrieval attempts, combined with contextual and dialogical constraints to narrow down the set of lexical candidates cued in the speech stream. Because of time constraints in dialogues, the listener may often settle for the gist without resolving all of the details of the signal-meaning mapping. It may even be the case that the context is so strongly predictive that very little if any information delivered by RAMBPHO is needed for successful recognition to occur (Moradi et al., 2013).

We now further assume that the information delivered by RAMBPHO is relayed by a fast-forward, matching mechanism that is nested under a slow, explicit feedback loop (cf. Poeppel et al., 2008; Stenfelt and Rönnberg, 2009). The mismatch in itself is determined either by poor RAMBPHO information and/or poor phonological representations in semantic LTM. We conceptualize the phonological representations in LTM in terms of multiple attributes. A minimum number of attributes are required for access to a certain lexical item. Above a certain threshold there is a sufficient number of attributes to trigger the lexical representation. Below threshold, we can expect a number of qualitatively different outcomes: (a) if the number of attributes is close to threshold, then some phonological neighbors may be retrieved (Luce and Pisoni, 1998); (b) if too few attributes match the intended target item, the matching process could be led astray by contextual constraints induced by “mismatching” semantic cues (Zekveld et al., 2011a); and (c), if no phonological attributes are present at the RAMBPHO level, it could still be the case that a sentence context is so predictive that an upcoming target word is very likely to be activated anyhow (Moradi et al., 2013).

The matching process is ultimately determined by the fidelity of the input and phonological representation. Fidelity is affected by external noise but also by internal noise (e.g., by poor phonological representations due to long-term hearing impairment; Classon et al., 2013a,b). These phonological attributes are primarily constrained at a syllabic level of representation. RAMBPHO information is based on rapid phonological extraction from the signal by means of a mix of visual, sound-based and motoric predictions (cf. Hickok, 2012). We still propose that the bottleneck of the system is the connection between RAMBPHO delivered information and the phonological-lexical representation. However, we now also assume that the phonological attributes are embedded within domains of semantically related attributes; i.e., relations between the two types of attributes are assumed to be stored and represented together (cf. Hickok, 2012). Thus, these synergistic representations allow lexical access both via RAMBPHO and semantic cueing (Zekveld et al., 2011a, 2012), and give ground for a conceptualization of a versatile, multi-code capacity usage of high WMC participants. Furthermore, in the new ELU model, the implicit as well as the explicit processing mechanisms rely on phonological and semantic interactions. Semantic LTM can be used either for explicit “repair” of a distorted signal, for inference-making, or for implicit and rapid semantic priming. Mismatch will determine how time is shared between the explicit and implicit operations: the fast (implicit) RAMBPHO mechanism is always running until it is temporarily interrupted. When interrupted, the default situation is that it re-starts the analysis of the speech signal with whatever information is available (phonological/semantic). At the same time, mismatch will tune the system to use the explicit slow loop to repair violated expectations, again via semantic and phonological cues.

Under time pressure, and given that the listener is happy to settle for the gist of the message (see Pichora-Fuller et al., 1998), low-level RAMBPHO processing may be overruled by explicit functions. RAMBPHO is in principle “blind” to the overall meaning of a message, in the sense that its sole function is to “unlock” the lexicon. But it is conceivable that it can be modified in terms of attention to certain attributes depending on semantic knowledge about speaker identity and topic (Mesgarani and Chang, 2012). The crucial aspect is therefore not the specific kind of signal processing that temporarily interrupts RAMBPHO, but the modality-general explicit capacity to use and combine the available perceptual evidence and quality of the LTM knowledge. This takes place via different WMC-dependent executive mechanisms such as inhibition, focusing of attention, and retrieval of contextual and semantic information. The sooner the brain can construct an interpretation of the message, the easier language processing becomes, and the content of a dialogue is more rapidly committed to more permanent memory encodings.

In short, the new ELU model is a WMC-based model of a meaning prediction system (cf. Samuelsson and Rönnberg, 1993; Federmeier, 2007; Hickok, 2012). Specifically, the settings of the system are regulated either explicitly (by some semantic/contextual instruction or explicit feedback) or by the neural consequences of high WMC (in terms of, e.g., brain oscillations). Attention manipulations—seen as one way of pre-tuning the system—have recently proven to have cortical consequences in speech in noise tasks (Mesgarani and Chang, 2012; Wild et al., 2012).

**Figure 2:**
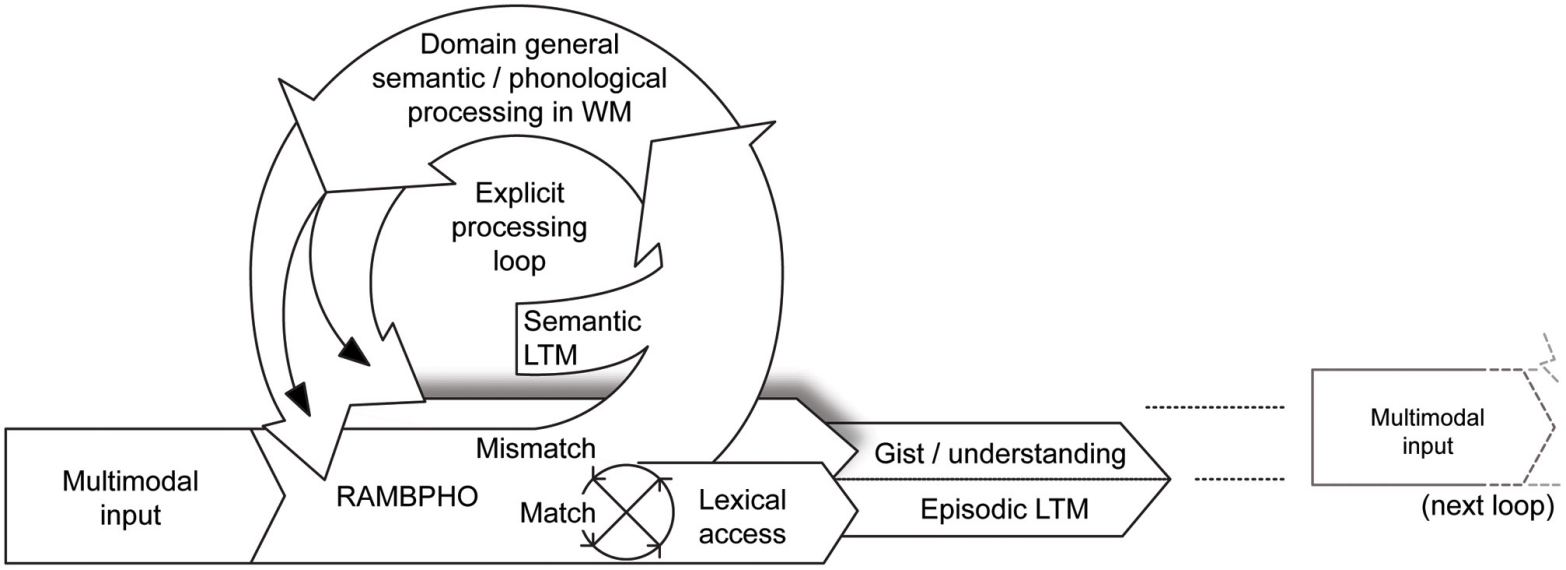
**The new Ease of Language Understanding (ELU) model.** In ideal listening conditions, multimodal RAMBPHO input matches a sufficient number of phonological attributes (i.e., above threshold) in the mental lexicon and lexical access proceeds rapidly and automatically. RAMBPHO may be preset by expectations—modulated by WM—concerning the phonological characteristics of the communicative signal, e.g., the language or regional accent of the communicative partner or by semantic or contextual constraints. When there is a mismatch (as in suboptimal listening conditions), WM “kicks in” to support listening (Rönnberg et al., 2010). The explicit, WMC-dependent, processing loop uses both phonological and semantic LTM information to attempt to fill in or infer missing information, which also feeds back to RAMBPHO. The output of the system is some level of understanding or gist, which in turn induces a semantic framing of the next explicit loop. Another output from the system is episodic LTM, where information encoded into LTM is dependent on the type of processing carried out in WM. Explicit and implicit processes run in parallel, the implicit being rapid, the explicit is a relatively slow feedback loop.

In Figure 2, we illustrate how explicit/implicit processes interact over time. Each explicit “loop” is activated by a mismatch. The number of times the listener passes through an explicit loop depends generally on for example turn taking, competing speech, attention manipulations, or to distortions from signal processing in the hearing aid.

## New ELU predictions

We outline some new predictions that follow from the revised and updated ELU model.

1. Signal distortion will tax WMC during speech understanding. This applies to different kinds of signal compression algorithms used in hearing aids, and to other kinds of distortion (cf. Foo et al., 2007; Arehart et al., 2013). Even at favorable SNRs, WMC modulates the effect of signal processing on short-term retention of spoken materials (Ng et al., 2013a). Still, effects relating to intended distortion of the target signal per se and the unwanted artifacts of signal processing (e.g., “musical noise” during binary masking) need to be teased apart.
2. WMC is predicted to modulate early attention mechanisms (Sörqvist et al., 2012a; cf. Kraus and Chandrasakaren, 2010; Kraus et al., 2012) and semantic framing (priming).
3. Classon et al. (2013a,b) demonstrated that WMC can compensate for phonological deficits. It also modulates the use of semantic cues during speech-in-noise understanding (Zekveld et al., 2012). It addition, it predicts facilitation of encoding operations (and subsequent episodic LTM) in conditions of speech-in-speech maskers (Sörqvist and Rönnberg, 2012). In short, participants with high WMC are predicted to better adapt to different task demands than participants with low WMC, and hence are more versatile in their use of semantic and phonological coding and re-coding after mismatch.
4. STM, and by inference, WM, is predicted to be more robust than LTM systems in response to impairment-related decline (Rönnberg et al., 2011a,b). This prediction should be further tested with different tasks assessing different memory systems before definite conclusions can be made.
5. WMC is related to effort (Koelewijn et al., 2012a,b), especially to intermediate levels of effort (Rudner et al., 2012). Further work is needed to uncover underlying mechanisms.
6. Predictions for sign language understanding should focus on visual noise manipulations and on semantic maskers to assess the role of WMC in understanding sign language under challenging conditions (Rönnberg et al., 2004; Rudner et al., 2007; Cardin et al., 2013). By testing whether WMC is also invoked in conditions with visual noise, the analogous mechanism to mismatch in the spoken modality could be evaluated.

### Relation to other models

The new ELU model differs from models of speech perception (e.g., the TRACE model, McClelland and Elman, 1986; the Cohort model, Marslen-Wilson, 1987; and the NAM model, Luce and Pisoni, 1998) and also from the original notion of mismatch negativity (Näätänen and Escera, 2000) in its assumption that explicit WMC is called for whenever there is mismatch between language input and LTM representations. In this way, the mismatch mechanism—and the demand on WMC—is related to communication. Nevertheless, the ELU model is similar to the earlier speech perception models in that all acknowledge the importance of an interaction with LTM representations and that lexical access proceeds via some kind of model-specific retrieval mode. The ELU model especially focuses on how the perceptual systems interact with different memory systems. The cognitive hearing science aspect and the historical context of the ELU model has recently been reviewed elsewhere (Pichora-Fuller and Singh, 2006; Arlinger et al., 2009).

RAMBPHO focuses on the integration of phonological information from different sources and thus shares similarities with the episodic buffer introduced by Baddeley (2000). However, unlike Baddeley's model, the ELU model is geared toward the communicative outcome, i.e., language understanding, rather than WMC as such (Rudner and Rönnberg, 2008a,b). The fact that the need for explicit resources such as WMC is restricted to mismatch situations also represents a unique processing economy aspect of the ELU model.

The ELU model is inspired by the notions and models of WM for read text presented by Daneman and Carpenter (1980) and Just and Carpenter (1992) in that it emphasizes both storage and processing components of WM. This is why we originally adopted the reading span task as a potentially important predictor variable of speech-in-noise performance, without introducing audibility problems. The trade-off between storage and processing is particularly relevant for the ELU model in that hearing impairment typically puts extra pressure on the processing and inference-making that is needed to comprehend a sentence. Less storage and less encoding into episodic LTM are expected for participants with hearing impairment compared to participants without hearing impairment unless they have a high WMC. Of particular relevance is the fact that Just and Carpenter (1992) showed that WMC constrains sentence comprehension during reading such that individuals with high WMC are better than individuals with low WMC at coping with more complex syntactic structures (e.g., object-relative clauses), maintaining ambiguous representations of sentences, and resolving anaphora. Dealing with semantic or syntactic complexity is presumably very important for participants who are “mismatching” frequently during conversation. Here, the attention, inference-making, inhibition and storage abilities of individuals with high WMC play a crucial role.

The ELU has some interesting similarities with the speech perception model by Poeppel et al. (2008) in that both models assume parallel processes (streams) that operate within different time-windows (cf. Hickok and Poeppel, 2004, 2007). For ELU the first time window is when phonological representations are formed in RAMBPHO to match representations in semantic LTM; the second is the slower explicit loop function. RAMPBHO seems to be a concept very similar to the phonological primal sketch suggested by Poeppel et al., where syllables mediate lexical access. Also, audiovisual integration seems to occur around 250 ms, where visual information typically leads and affects the integration (van Wassenhove et al., 2007). We have speculated about the earlier (than syllabic) spectral-segmental kinds of analyses discussed in the Poeppel et al. (2008) paper (see Stenfelt and Rönnberg, 2009), primarily in terms of how different types of hearing impairment might affect perception of segmental features.

The explicit, slow processing loop, is *postdictive* in the sense that mismatch, error-induced, signals may invoke some kind of WM-based inference-making. This was the main function for re-construction and inference-making in the old ELU model (Rönnberg, 2003; Rönnberg et al., 2008). However, as we have emphasized with the new ELU model, its *predictive* potential is now clearly spelled out in terms of the re-settings explicit processes may invoke, phonologically and semantically, and also because of the fine-tuning or synchronization by WM itself (Hickok, 2012). In keeping with Poeppel et al. (2008), the neural basis of syllabic processing is likely to involve STS, lexical access supposedly involves MTG, while our recent study (Zekveld et al., 2012) is a first indication of a frontal (LIFG) WMC-based compensation for the explicit effort involved in decoding words/sentences in noise. Poeppel et al. (2008) advocate an analysis by synthesis framework whereby initial segments of a spoken signal are matched against a hypothesis, “an internal forward model.” The internal model is then updated against new segments of speech approximately at every 30 ms, feeding back to several levels of representation including the phonological primal sketch. One way of conceptualizing the hypothesis-driven, analysis-by-synthesis framework by Poeppel et al. (2008) may in fact be understood in terms of WMC. A high WMC helps keep several hypotheses alive, allowing for top-down feed-back at several points in time and at segmental, syllabic, lexical and semantic levels of representation (cf. Figure 4 in Poeppel et al., 2008, cf. Poeppel and Monahan, 2011). The probability of entertaining or maintaining a hypothesis in WM may then in part be determined by Bayesian logic, “The quantity p(H|E) represents the likelihood of the hypothesis, given the sensory analysis; p(E|H) is the likelihood of the synthesis of the sensory data given the analysis” (p. 1080, Poeppel et al., 2008), where H represents the forward hypothesis and E the perceptual evidence. With an ELU perspective, this will also be modulated by the WMC to hold several hypotheses, at different levels in the cognitive system, in mind.

In the general context of dual stream models, addressing the interaction between ventral and dorsal attention networks, Asplund et al. (2010) found that so called surprise blindness, i.e., where a profound deficit in the detection of a goal-relevant target (a letter) as a result of the presentation of an unexpected and task-irrelevant stimulus (a face), causes activity in the inferior frontal junction. This manipulation represents an interaction between stimulus-driven and goal-directed, hypothesis-driven attention and may be compared to the cueing manipulations by Zekveld et al. (2011a, 2012). Resolutions of ambiguity also involve interactions between stimulus-driven and knowledge-driven processes (Rodd et al., 2012), which demand the integrative functions of LIFG. These examples may in fact be related to the new predictive and postdictive (feedback) interactions postulated in the new ELU model.

As discussed by Arnal and Giraud (2012), implicit temporal predictions of spoken stimuli represent one mechanism that may be modulated by slow delta-theta oscillations, whereas in the case of top-down, hypothesis-driven transmission of content specific information, beta oscillations may index a complementary mechanism in speech comprehension. Similar kinds of dual mechanisms have been proposed by Golumbic et al. (2012) when tracking selective attention to a target voice while ignoring another voice in a cocktail party situation. Low frequency activity typically corresponds to the speech envelope at lower auditory cortex levels, whereas high gamma power activity is reflected in the entrainment to the attended target voice only at later stages of processing, which also were cortically spread out to, e.g., inferior frontal cortex and anterior temporal cortex. This general result connects nicely with the Zekveld et al. (2012) data of WMC based compensation localized in LIFG and MTG.

Finally, Andersson and colleagues demonstrated in a recent study (Anderson et al., 2013a), using structural equation modeling, that auditory WM, in combination with central auditory functions such as brain stem responses (e.g., pitch encoding), contributes to understanding speech in noise. Peripheral auditory measures *did not* account for any variance but musical experience reinforced the effect of auditory WM. This is in line with our research ascribing a central role to WM for speech understanding under adverse conditions. Interestingly, Anderson et al. (2013b) have also been able to show that brain stem responses to complex sounds, rather than hearing thresholds, predict self-reported speech-in-noise performance. These data agree with the Sörqvist et al. (2012a,b) data on the relationship between brain stem responses and WMC. Since WM is by definition an explicit processing and storage system, the data also fit with the fact that self-report—which taps into explicit awareness of speech processing (cf. Ng et al., 2013b)—has the capacity to reflect brain stem responses.

In sum: although the ELU model shares underlying notions with other speech perception and WM models, its uniqueness lies in the connection between mismatch and WMC (explicitly and postdictively), and implicitly and predictively, between WM and RAMBPHO, and the roles played by the interaction between WM and other memory systems such as episodic and semantic LTM.

### Limitations

One limitation of the new ELU model concerns the more exact definition of when a mismatch condition is at hand. We have seen a picture of results that suggests that many kinds of signal processing actually demand a higher dependence of WMC, at least initially, before some learning or acclimatization has occurred (cf. the first prediction). This of course also holds true for the case when a person has acclimatized to a certain kind of signal processing, and then is tested with another, thus breaking, the habitual phonological coding schemes. However, a critique that can be launched is that we a priori may have problems determining the exact parameters for the mismatch induction. The problem of circularity is apparent. More empirical investigations into, e.g., determining whether it is the kind of signal processing or the artifacts caused by signal processing that determine mismatch and WM dependence will help clarify this issue. Another problem relates to the (so far) relatively few studies involving the neural correlates of WMC and speech understanding in noise. Future studies will also have to address the neural consequences of high vs. low WMC and how it modulates predictions at different linguistic levels (syllabic, lexical, semantic, and syntactic).

## Clinical implications

Given that WMC is crucially important for on-line processing of speech under adverse conditions as well as the ability to maintain its content for shorter or longer periods, then hearing aid manufacturers, speech-language pathologists and hearing health care professionals must take that into account. First, clinically relevant WMC tests need to be developed; tests that tap into the processes that have proven to be modality-general and optimal for both on-line processing of speech as well as for episodic LTM. This means normative data needs to be collected to determine age-dependent and impairment-specific performance levels and provide a clinical instrument for assessing WMC. By using visual-verbal tests audibility problems are avoided, thus disentangling potential perceptual degradation effects from WM performance. However, it is important to collect norms for different age-groups and levels of hearing impairment in combination, because there is also the possibility of more central, or cognitive side-effects of age and impairment.

Second, individuals with low WMC seem to be initially susceptible to signal processing distortions from “aggressive” signal processing (fast amplitude compression, severe frequency compression, binary masking), although this susceptibility may decline after a period of familiarization (Rudner et al., 2011). For all individuals, concrete options are at hand for manipulations of the signal in the hearing aid: to increase amplification, alter input dynamics, to remove some information (= noise reduction) to get a benefit. But these manipulations come at a cost that is different for different individuals. Thus, we advocate that the “dose of the medicine” (= the active ingredient), the intended benefit of signal processing and its side-effects (by-product of the medicine) must be tailored to the individual, such that the high WMC can have a more active ingredient (= more aggressive signal processing, compared to the low WMC who may be more susceptible to side-effects). This reasoning could in principle also be applied to acoustic design of other technologies.

Third, the data we have presented suggest that many kinds of more advanced signal processing in hearing instruments demand WMC. The down-side of using advanced signal processing on a daily basis is that it demands effort and for any given individual with hearing loss, this may outweigh the benefit. Therefore, there is a need to develop new methods that assess effortful brain-work with more precision. Here, reaction time measures, pupil dilation indices or measures of evoked response potentials may prove to be useful signals for on-line adjustment of signal processing parameters in hearing instruments.

Fourth, with a new cognitive hearing science perspective, it would be equally important to evaluate memory and comprehension of the contents of a conversation in noise, as functional outcome measures, rather than only focusing on word recognition accuracy per se (Pichora-Fuller, 2007; Rönnberg et al., 2011a). This can actually be seen as an indirect measure of cognitive spare capacity, or the residual cognitive capacity that remains once successful listening has taken place (Pichora-Fuller, 2007, 2013; Mishra et al., 2013).

Fifth, it is quite possible that to properly evaluate the effects of hearing aids and other interventions, a longitudinal study that also systematically manipulated the kind of signal processing would be quite informative. We know very little about the long-term effects of signal processing on cognition and how this may relate to or reduce the risk of dementia (Lin et al., 2011, 2013).

Finally, an intervention study that evaluated the effects of WM training on speech in noise understanding would put the causal nature of WMC to the test (cf. McNab et al., 2009). Additionally, if one could study the neural correlates of this putative plastic change that would shed further light on the neural mechanisms involved.

### Conflict of interest statement

The authors declare that the research was conducted in the absence of any commercial or financial relationships that could be construed as a potential conflict of interest.
